# Aspirin Combined with Antifungal Drugs Suppresses *Candida albicans* Biofilm by Activating Autophagy

**DOI:** 10.4014/jmb.2411.11060

**Published:** 2025-03-27

**Authors:** Yun Huang, Haochen Miao, Ying Lv, Yang Wang, Shenjun Yu, Xin Wei

**Affiliations:** 1 Department of Endodontics, The Affiliated Stomatological Hospital of Nanjing Medical University, Nanjing, P.R. China; 2 State Key Laboratory Cultivation Base of Research, Prevention and Treatment for Oral Diseases, Nanjing Medical University, Nanjing, P.R. China; 3 Jiangsu Province Engineering Research Center of Stomatological Translational Medicine, Nanjing, P.R. China

**Keywords:** *Candida albicans* biofilm, aspirin, autophagy, reactive oxygen species, mitochondrial dysfunction

## Abstract

Aspirin (ASA) induces autophagic death of human tumor cells and autophagy changes the susceptibility of *Candida albicans* biofilm to antifungal agents. This study investigates whether ASA suppresses *C. albicans* biofilm by autophagy regulation and its combination effect with antifungals. Biofilm sensitivity to ASA alone and in combination with antifungals was evaluated using the checkerboard method, and drug interactions were assessed by the fractional inhibition concentration index (FICI) and ΔE models. The effects of ASA on mTOR signaling were examined by western blotting. Alkaline phosphatase activity, acridine orange stain assay, and autophagy-related gene expressions were examined to evaluate autophagic activity. Autophagosomes were observed by transmission electron microscopy. Reactive oxygen species (ROS) were detected by DCFH-DA. Mitochondrial membrane potential (MMP), malondialdehyde (MDA), and ATP levels were determined using commercial kits. ASA inhibited *C. albicans* biofilm in a concentration dependent manner and showed synergistic effects against biofilms when combined with amphotericin B or 5-fluorocytosine. ASA treatment induced oxidative stress, evidenced by increased ROS and MDA levels, alongside a reduction in ATP and MMP. ASA inhibited mTOR signaling and induced autophagy in *C. albicans* biofilms by increasing oxidative stress and mitochondrial dysfunction, contributing to biofilm inhibition. This study provides valuable insights into the potential of ASA as an adjunct therapy in combination with antifungal agents for managing *C. albicans* biofilm-related infections.

## Introduction

*Candida albicans* is a clinically significant pathogen that is responsible for many superficial and systemic infections. *C. albicans* colonization is a risk factor for disease, especially in immunocompromised patients [1]. *C. albicans* forms biofilms comprised of yeast cells, pseudohyphae and hyphae. The transition from planktonic growth to biofilm is accompanied by a complex remodeling and *C. albicans* biofilms are highly tolerant to antifungal therapy and can serve as a reservoir for recurrent infection, which increases the difficulty of clinical treatment [2].

Aspirin (acetyl salicylic acid (ASA)), a widely used nonsteroidal anti-inflammatory drug (NSAID), has been shown to enhance the sensitivity of *C. albicans* to antifungal agents, particularly in strains resistant to fluconazole. ASA reduces *C. albicans* adhesion to abiotic surfaces and regulates the process of biofilm formation and viability in *C. albicans* [3]. In addition, ASA combined with amphotericin B enhances its inhibitory effect on *C. albicans* and ASA also lowers the minimum inhibitory concentrations of fluconazole and itraconazole against *C. albicans* [3, 4]. Also, there was a synergistic interaction between ASA and caspofungin against 16 out of 39 clinical strains of *C. albicans* biofilms. However, there have been no reports on the combination of ASA with 5-fluorocytosine and terbinafine, and currently, the evaluation method for analyzing the combined effect in relevant studies is only the FICI method [3]. The ΔE method does not rely on MIC values, complementing the shortcomings of the FICI method [5]. Therefore, this work evaluated the inhibitory effects of ASA and antifungal drugs on *C. albicans* biofilms, using the FICI and ΔE methods. ASA inhibits *C. albicans* biofilm formation by reducing prostaglandin E2 (PGE2) synthesis, but further research suggests its effects are not fully mediated by PGE2 [6, 7]. In studies where both NSAIDs and PGE2 were added to *C. albicans* biofilms, PGE2 completely reversed the inhibitory effects of other NSAIDs such as sodium salicylate and indomethacin. However, PGE2 only partially reversed the effects of ASA [7]. These findings suggest that ASA may act through additionally, yet undefined, molecular targets or mechanisms. Further studies are necessary to elucidate the specific pathways involved in ASA’s inhibition of *C. albicans* biofilms.

Studies showed that autophagy changed the susceptibility of *C. albicans* biofilms to antifungal agents [8]. Autophagy is a core molecular pathway for the preservation of cellular and organismal homeostasis and has also been shown to regulate the formation of biofilms in *C. albicans* [9-11]. ASA can inhibit mTOR signaling, induce autophagic death and inhibit their proliferation of various human tumor cells [12, 13]. The treatment of ASA in *Cryptococcus* led to stress induction and cell death was eventually achieved through ROS-mediated membrane damage [14, 15]. ROS could promote the formation of autophagy and autophagy, in turn, may contribute to reduce oxidative damage by engulfing and degradating oxidized substance [16]. Therefore, this experiment proposes a hypothesis that ASA can induce oxidative stress changes and activate autophagy in *C. albicans* biofilm, thereby inhibiting biofilm formation. Exploring the autophagy related molecular mechanisms of aspirin's inhibitory effect on *C. albicans* biofilm provides an experimental basis for identifying potential therapeutic targets.

## Materials and Methods

### Strains and Culture

Overnight cultures of the following microorganisms were used throughout the study: *C. albicans* SC5314 (ATCC MYA-2876TM). Long-term maintenance of the microbial strains was performed at −20°C using glycerol and short-term maintenance was on yeast extract peptone dextrose (YPD) medium (1%yeast extract, 2%peptone, 2%glucose, 2% agar) at 4°C.

Planktonic *C. albicans* culture: The strain was cultured at 30°C, 200 rpm overnight on YPD (OXOID Inc., USA, 1%yeast extract, 2% peptone, 2% glucose). The cells were harvested, washed twice with phosphate buffer saline (PBS, Vazyme Biotech Co.,Ltd., China), and resuspended in YPD medium with a cell concentration of 2.5 × 10^3^ cells/ml.

*C. albicans* biofilm development: Aliquots of 100 μl of standard cell suspensions (1.0 × 10^6^ cells/ml) in Roswell Park Memorial Institute (RPMI)-1640 medium (Gibco Ltd., UK) were transferred into 96-well microtiter plates and incubated at 37°C. After incubation for 2 h, non-adherent cells were removed by washing twice with PBS. The plates were then incubated for 6, 12, 24, and 48 h for the development of biofilms.

### Preparation of ASA and Antifungal Agents

The antifungals were dissolved in dimethylsulfoxide (DMSO) and prepared in serial twofold dilutions based on the protocol M27-A4 from the Clinical and Laboratory Standards Institute (CLSI) (2017). Stock solutions of each antifungal were initially prepared at a high concentration (*e.g.*, 512 μg/ml for fluconazole, 64 μg/ml for itraconazole, etc.) in DMSO. A series of twofold dilutions were then made by transferring 1 ml of the stock solution into a new tube containing 1 ml of appropriate diluent (powdered RPMI-1640 medium) for each subsequent dilution step. This process continued until the desired final concentrations were reached: 0.125-128 μg/ml for fluconazole, 0.0313-32 μg/ml for itraconazole, 0.0313-16 μg/ml for amphotericin B, 0.0625 to 32 μg/ml for nystatin, 0.015-8 μg/ml for caspofungin, 0.125-128 μg/ml for 5-fluorocytosine and 0.125-64 μg/ml for terbinafine (Sigma-Aldrich, USA). A stock solution of ASA (900 mg/ml, Sigma-Aldrich) was freshly prepared in DMSO to reach final concentrations ranging from 1-10 mg/ml. After preparing the antifungal and ASA solutions, each was filtered through a 0.22 μm sterile filter (Millipore, USA) to remove any particulate matter before use in the susceptibility assays.

### Drug Addition and Susceptibility Testing

For the preparation of *C. albicans* planktonic cultures, 100 μl of cell suspensions, adjusted to a concentration of 2.5 × 10^3^ cells/ml, was added to each well of a 96 well culture plate. Subsequently, 100 μl of the drug solution was added to each well, prepared in a gradient of concentrations (L-gradient). The plates were incubated at 37°C for 24 h. For *C. albicans* biofilms, at 24 h of incubation, the biofilms were washed twice with PBS, followed by the addition of 100 μl of RPMI-1640 medium. For the SMIC studies, 100 μl of either aspirin or each serially diluted drug was added and incubated for 24 h. The SMIC_50_ (Sessile Minimum Inhibitory Concentration 50%) was analyzed using XTT assay and absorbance was measured at 490 nm using a microplate reader (SpectraMax M2, Molecular Devices, USA). For the combination studies, checkerboard assay was used to evaluate the antimicrobial efficacy of ASA and antifungal drugs upon combination as described above.

### Time-Growth Curve Experiment

Different concentrations of ASA were added to *C. albicans* biofilms (24 h) and the mixture was incubated at 30°C, 200 rpm for 24 h. Absorbance was measured at 630 nm after dosing using a microplate reader and a curve was drawn to record the growth of *C. albicans* at each time point.

### Time-Kill Curve Experiment

To further investigate the inhibitory effect of serially diluted ASA alone or combined with antifungal drugs on *C. albicans* biofilm, we performed a time-kill test *in vitro*. Biofilms (24 h) in 96-well microtiter plates were developed as described previously. The biofilms were washed twice with PBS, followed by the addition of 100 μl of RPMI-1640 medium. ASA alone, antifungal drugs alone, or a combination of ASA and antifungal drugs were added, and the mixtures were incubated at 37°C. At predetermined time points (6 h, 24 h, and 48 h), XTT assay was performed to detect cell viability in the biofilm.

### Drug Interaction Modeling

Even when the same methodology is used for testing the *in vitro* susceptibilities to drug combinations variable conclusions might be inferred depending on the way data are analyzed. Therefore, the data obtained were analyzed using two different models, FICI and ΔE, that have been used to characterize antifungal drug interactions [3, 5].

FICI: The interactions between antifungal agents (drugs A and B) were quantitatively evaluated by the FIC index, which was calculated according to the formula (MIC of A in combination/MIC of A)+(MIC of B in combination/MIC of B). If the FIC index was≥4 is usually considered antagonism, and if it was≤0.5 is synergy. The FICI method is widely used to interpret the interaction between antifungal drugs due to its simplicity, strong operability, and good reproducibility. However, the FICI model for assessing drug interactions has several limitations, including issues with result summarization, statistical interpretation, and inaccurate FICI values when off-scale MICs are present.

ΔE model: ΔE is a nonparametric model based on the BI theory. The BI theory is described by the equation Ii = (IA + IB) − (IA × IB), where Ii is the predicted percentage of inhibition of the theoretical noninteractive combination of the drugs A and B and IA, and IB are the experimental percentages of inhibition of each drug acting alone, respectively. Since I = 1 − E, where E is the percentage of growth, by substituting into the former equation, the following equation is derived: Ei = EA × EB, where Ei is the predicted percentage of growth of the theoretical noninteractive combination of the drugs A and B, respectively, and EA and EB are the experimental percentages of growth of each drug acting alone, respectively. Interaction is described by the difference (ΔE) between the predicted and measured percentages of growth with drugs at various concentrations. Because of the nature of interaction testing using microtiter plates with 2-fold dilutions of either drug, this results in a ΔE value for each drug combination. Unlike the FICI method, the ΔE model does not depend on MIC values, avoiding potential errors associated with MIC-based calculations. Additionally, the ΔE model provides a more comprehensive representation of drug interaction results. Each drug combination can be visualized using intuitive two-dimensional and three-dimensional plots, with the ΔE value depicted on the z-axis to create a surface plot. This method enhances the clarity and interpretability of the data. However, the ΔE model requires more complex calculations and careful interpretation compared to the FICI method.

In this study, a “relevant effect” was defined as either a synergistic interaction (FICI ≤ 0.5 or ΔE > 0) or an antagonistic interaction (FICI > 4 or ΔE < 0).

### Membrane Integrity Assay Using Propidium Iodide (PI) Staining

To evaluate the impact of ASA and 5-fluorocytosine on membrane integrity, *C. albicans* biofilms cells were treated with ASA, 5-fluorocytosine, or their combination at the concentration of SMIC_50_ for 24 h. Untreated cells served as the control. After treatment, cells were incubated with PI dye (PI:PBS = 1:1000) at room temperature for 30 min in the dark. Fluorescence was measured using inverted fluorescence microscope (DMI 3000B, Leica, Germany).

### Reactive Oxygen Species (ROS) Production

The dye 2’,7’-dichlorofluorescein diacetate (DCFH-DA) from Sigma Chemicals (USA) was used to detect the generation of intracellular ROS. *C. albicans* biofilms (24 h) treated with ASA at the concentration of SMIC_50_ for 24 h were collected and stained with DCFH-DA at a final concentration of (10 μM). After 30 min of incubation at 30°C. Mean fluorescence intensity (excitation: 488 nm and emission: 525 nm) of the cells was measured with a microplate reader (SpectraMax M2, Molecular Devices, USA) and observed using inverted fluorescence microscope (DMI 3000B, Leica).

### Measurement of Mitochondrial Membrane Potential (MMP)

A JC-1 kit (Beyotime Biotechnology, China) was used to analyze changes in MMP. *C. albicans* biofilms (24 h) were treated with ASA at the concentration of SMIC_50_ for 24 h, biofilms of each group were then stained with JC-1 at 37°C for 20 min in the dark. JC-1 fluorescence (green and red) was observed by an inverted fluorescence microscope (Leica DMI3000B). JC-1 exhibits red fluorescence in healthy mitochondria with high membrane potential and green fluorescence in depolarized mitochondria with low membrane potential. Monitoring both green and red fluorescence allows for assessment of changes in mitochondrial membrane potential [8].

### MDA Assay

Lipid peroxidation was quantified based on the MDA assay. *C. albicans* biofilms (24 h) were treated with ASA at the concentration of SMIC_50_ for 24 h, and each sample was processed according to the instructions of MDA reagent kit. Absorbance of the reaction mixture was measured at 532 nm using a spectrophotometer.

### ATP Synthesis Assay

The cellular ATP level was detected using an ATP Bioluminescence Assay Kit (Beyotime Biotechnology Co.). *C. albicans* biofilms (24 h) were treated with ASA at the concentration of SMIC_50_ for 24 h and incubated with vehicle or different concentrations of BN-3b (25.0, 50.0, and 75.0 μg/ml) for 8h. After incubation, cells from each culture were lysed and centrifuged. 100 μl of ATP detection working solution and 50 μl supernatant were mixed, and then luminescence was measured on a microplate reader at 636 nm.

### Alkaline Phosphatase (ALP) Activity Assay

*C. albicans* biofilms treated with ASA at the concentration of SMIC_50_ for 24 h were collected for ALP activity assay. ALP activity was examined using Phosphatase Assay Kit (Beyotime Biotechnology Co., Ltd.) and the absorbance was obtained at 405 nm using a microplate reader (Thermo Fisher Scientific Inc., USA). The results were normalized to the total intracellular protein content determined by the Bradford Kit (Beyotime Biotechnology Co.) and expressed in nanomoles of produced p-nitrophenol per min per mg of protein (nmol/min/mg protein). The assay was repeated at least three times per strain.

### Acridine Orange (AO) Stain Assay

*C. albicans* biofilms treated with ASA at the concentration of SMIC_50_ for 24 h were collected for AO stain assay. The AO stain was carried out using AO Detection Kit (Solarbio Science & Technology Co. Ltd., China). After adding the lysing enzyme to the samples and mixing for 15 min, the AO staining was added according to the proportion of 1:1 and incubated at 37°C for 30 min. The cells were collected and mixed with PBS, and the staining percentage was finally detected using BD FACSCalibur flow cytometer (Biosciences Co. Ltd., China). The assay was repeated at least three times per strain.

### Analysis of Cell Morphology

The biofilm morphologies were observed by scanning electron microscopy (SEM). *C. albicans* biofilms treated with ASA at the concentration of SMIC_50_ for 24 h were collected and fixed in 2.5% (v/v) glutaraldehyde overnight at 4°C. Biofilms were dehydrated using a graded ethanol series: 30%, 50%, 70%, 90%, and 100% ethanol, each step lasting 10 min. After dehydration, the biofilms were put into a critical point dryer (Leica model EM CPD300, Austria) for drying, gold coated for 55 s at 45 mA using a SPI Module-Sputter Carbon/Gold Coater (West Chester, USA), and imaged using a SEM (1530VP, LEO, Germany). The imaging was performed at a magnification of ×1,000, ×2,000, and ×5,000, with an accelerating voltage of 20 kV, the beam current of 1.5 nA for sample examination and 50 pA for image acquisitionn.

The effect of ASA on the ultrastructure of *C. albicans* biofilm cells was analyzed using Transmission electron microscopy (TEM). *C. albicans* biofilms (24 h) treated with ASA at the concentration of SMIC_50_ for 24 h were collected. After fixation and dehydration, samples were impregnated before being embedded in epoxy and acrylic resins and then cut into 60 nm in thickness using an ultramicrotome. Samples were observed under a Hitachi H-7650 TEM (Japan).

### RNA Extraction and RT-*q*PCR

The approaches used for RNA extraction and RT-*q*PCR were performed as previously described [17]. The mRNA levels of the target genes were measured through an ABI 7900 Fast Real-time PCR machine (Applied Biosystems, Switzerland) using AceQ qPCR SYBR Green Mix (Vazyme, China), according to the manufacturer’s instructions. For relative mRNA quantification, the 2^-ΔΔCt^ method was used, with ACTIN as the internal control. All PCR primers used here are listed in Table 1. The test was repeated at least three times per strain.

### Western Blotting

Total protein extracts of *C. albicans* were prepared from an immunoprecipitation protocol as previously described [18]. Equal amounts of protein per lane were separated by 10% SDS-PAGE and transferred onto a polyvinylidene difluoride membrane (PVDF, Cat# IPVH00010, Millipore, China), blocked with 5% skimmed milk (BD Difco, Cat# 232100, USA) in PBS for 2-3 h with gentle shaking. Then, the membranes were incubated with related primary antibodies the membranes were incubated with related antibodies including β-ACTIN (1:8000), GAPDH (1:10000), Atg6 (1:1000), Atg7 (1:1000), Atg13 (1:1000), Atg27 (1:1000), and phosphorylated and non-phosphorylated S6 (1:800) overnight at 4°C. After being washed three times with 0.1% Tween-20 in Tris-buffered saline (TBST) buffer, the membranes were incubated with horseradish peroxidase-conjugated anti-rabbit secondary antibodies (1:10000, SA00001-2, Proteintech) for 1 h at room temperature with gentle shaking. Repeat the above-washed steps. Finally, the proteins were visualized with enhanced chemiluminescence reagent (Cat# HP5002, Noblebio, Nederland), imaged by the chemiluminescence system (Merck & Co., Inc., USA). Relative protein levels were quantified using ImageJ software (National Institutes of Health, USA), and the gray value ratio was used for normalization.

### Statistical Analysis

Data are presented as the mean±standard deviation (Mean ± SD) of multiple experimental repetitions. Statistical analysis and graphical representation were conducted using GraphPad Prism 8 (GraphPad Inc., USA). Differences between the two groups were analyzed using Student’s *t*-test. A *p*-value of <0.05 was considered statistically significant.

## Result

### Minimum Inhibitory Concentrations (SMICs) of ASA and Antifungal Drugs in *C. albicans* Biofilm

**Fig. 1:**
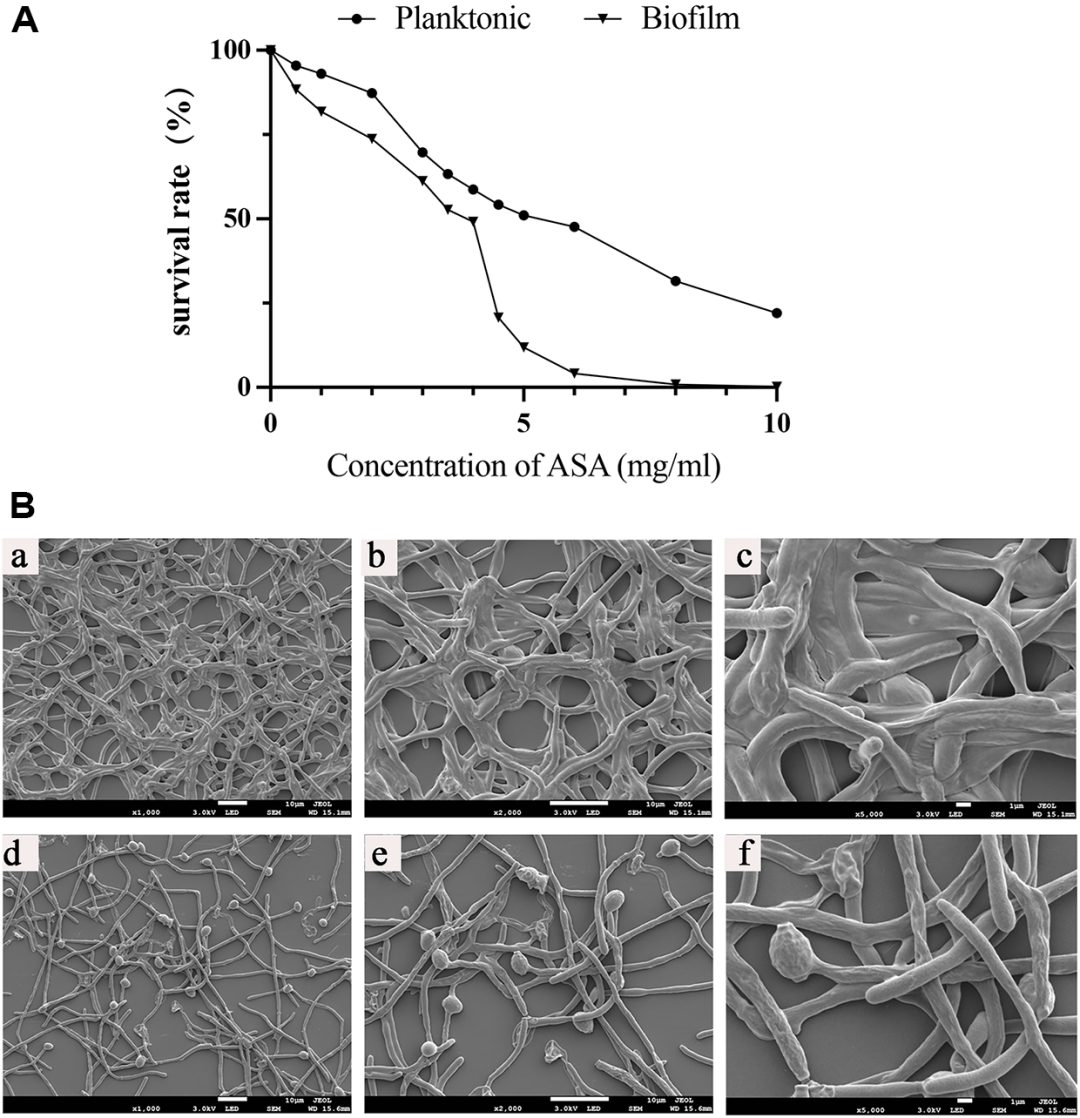
Effect of ASA on *C. albicans*. (**A**) The survival rate of curves of planktonic *C. albicans* and *C. albicans* biofilm treated with ASA; (**B**) Morphological changes of *C. albicans* biofilm (24 h) after the addition of ASA (4 mg/ml) by SEM, (a-c) Control; (d-f) ASA.

Within the concentration range of 4–10 mg/ml, ASA has significant inhibitory effects on both planktonic *C. albicans* and *C. albicans* biofilm. It was observed that under biofilm condition the SMIC_50_ of ASA was 4 mg/ml and the inhibitory effect of ASA on *C. albicans* biofilm continuously increases with the increase of ASA concentration, indicating that the inhibition of on *C. albicans* biofilm by ASA is concentration dependent (Fig. 1A). SEM observation indicated that ASA induced significant changes in biofilm microstructures (Fig. 1B). Specifically, ASA-treated biofilms exhibited a marked reduction in hyphal structures and an increase in yeast-form cells (Fig. 1B). Such structural disruptions indicate that ASA not only inhibits biofilm formation but also impacts the overall morphology and structural integrity of *C. albicans* biofilms. The SMIC_50_ of antifungal drugs were showed in Table 2.

### Assessment of In Vitro Interaction between ASA and Antifungal Drugs against *C. albicans* Biofilm Using the FICI and Δ E Method

**Fig. 2:**
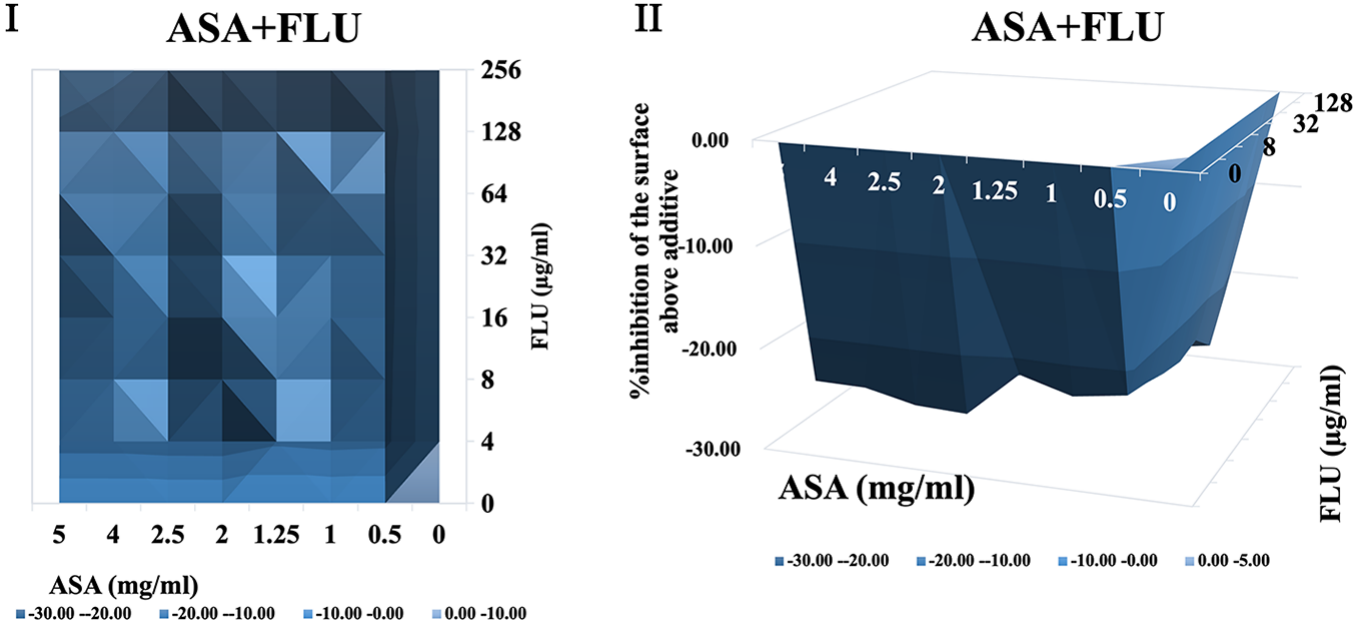
The contour (I) and three-dimensional (II) plots of interaction between ASA and fluconazole (FLU) against *C. albicans* biofilm.

**Fig. 3:**
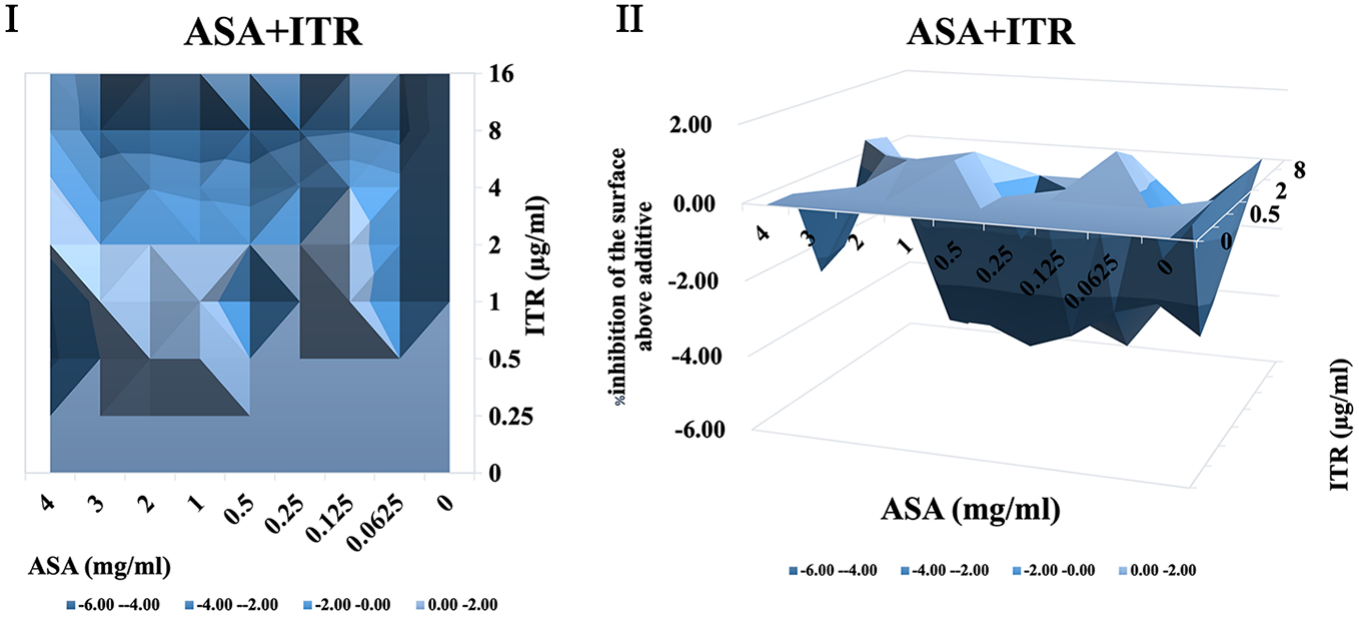
The contour (I) and three-dimensional (II) plots of interaction between ASA and itraconazole (ITR) against *C. albicans* biofilm.

**Fig. 4:**
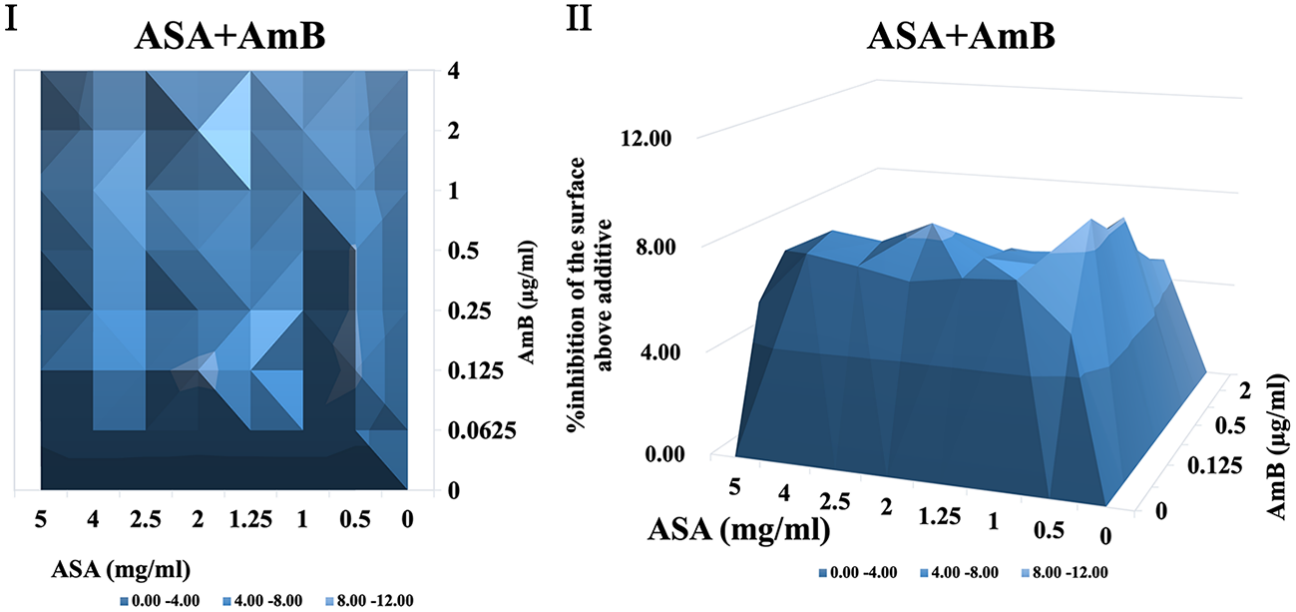
The contour (I) and three-dimensional (II) plots of interaction between ASA and amphotericin B (AMB) against *C. albicans* biofilm.

**Fig. 5:**
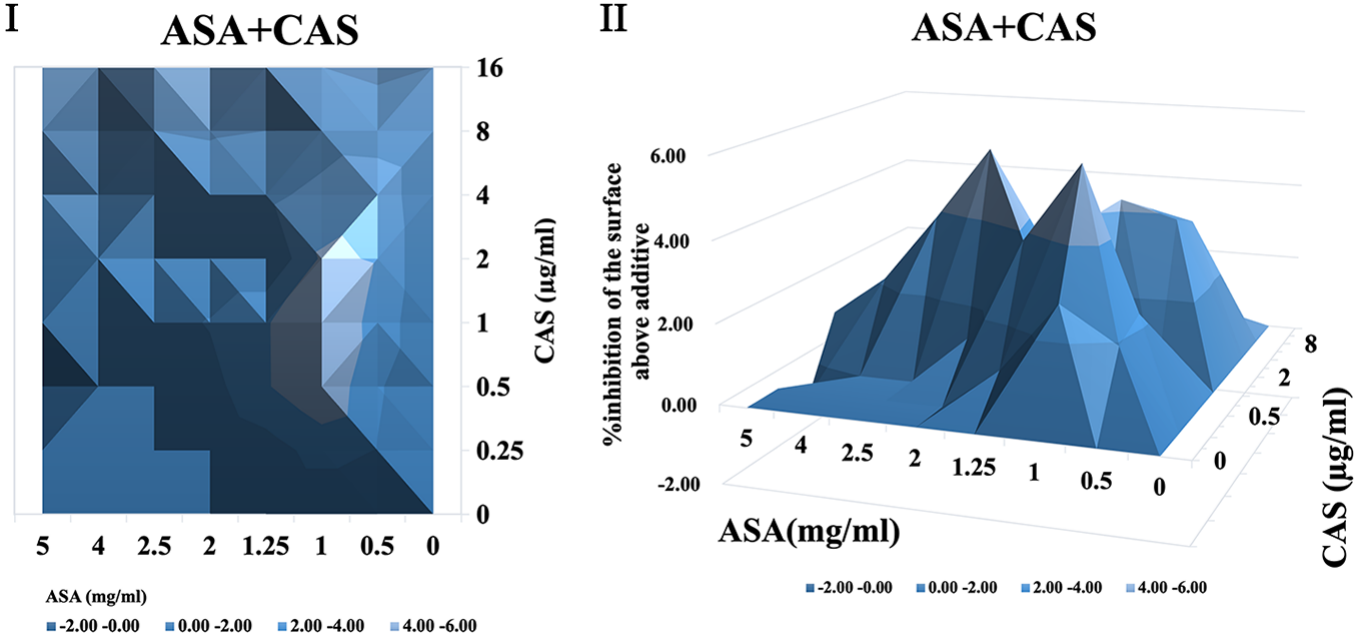
The contour (I) and three-dimensional (II) plots of interaction between ASA and caspofungin (CAS) against *C. albicans* biofilm.

**Fig. 6:**
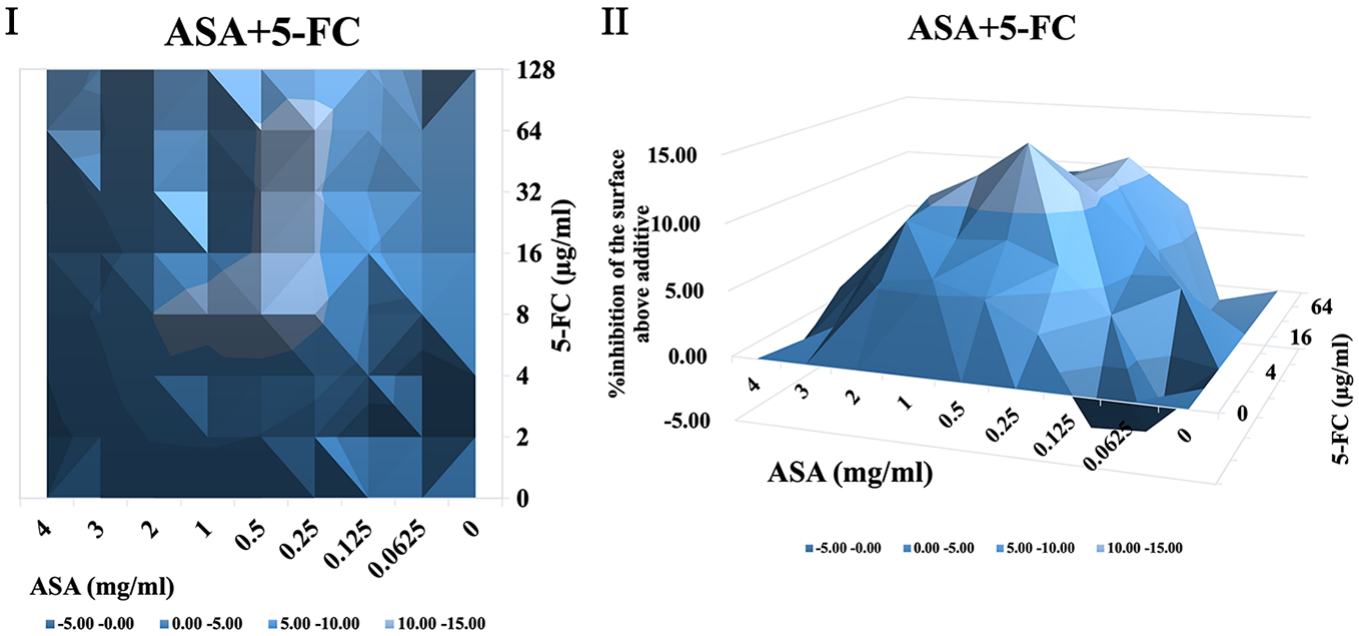
The contour (I) and three-dimensional (II) plots of interaction between ASA and 5-fluorocytosine (5- FC) against *C. albicans* biofilm.

**Fig. 7:**
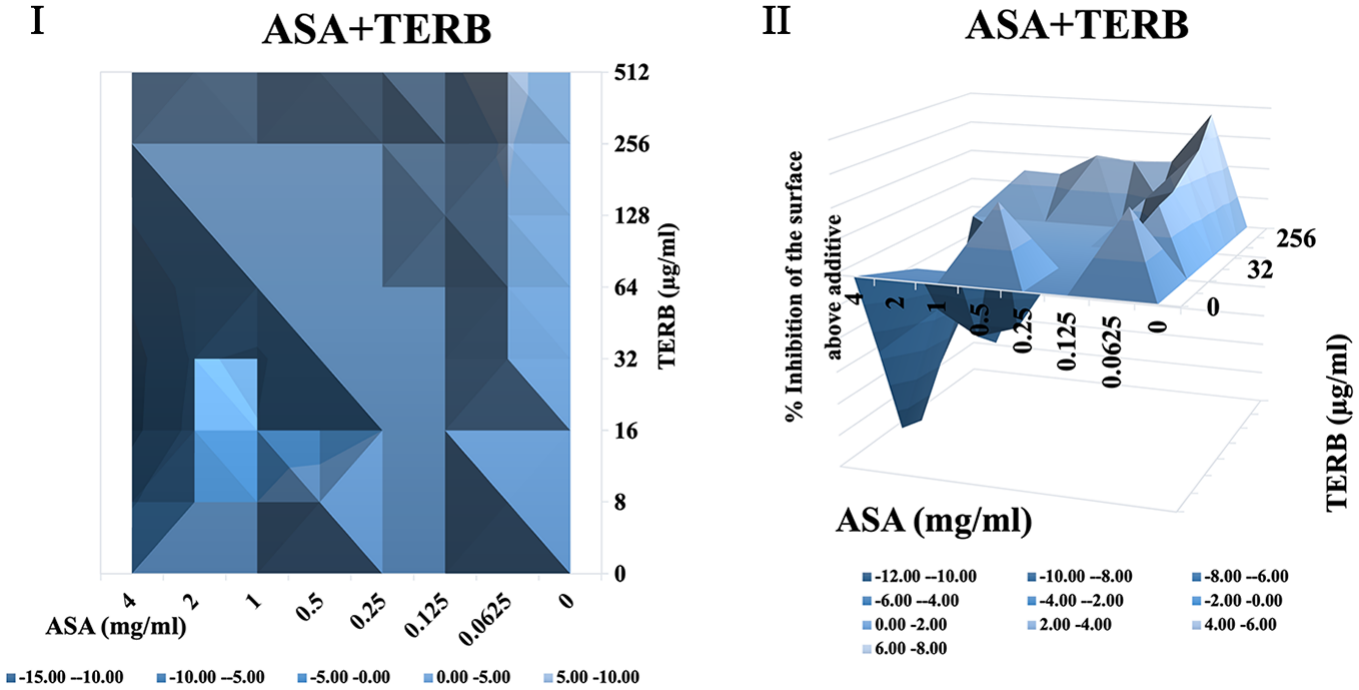
The contour (I) and three-dimensional (II) plots of interaction between ASA and terbinafine (TERB) against *C. albicans* biofilm.

It was shown that when treated with ASA combined with amphotericin B or 5-fluorocytosine, synergistic interactions were observed in *C. albicans* biofilm using both FICI and Δ E method. The contour and three-dimensional plots for these combinations revealed clear regions of synergy, indicated by positive ΔE values at specific concentration ranges (Figs. 4 and 5). When treated with ASA combination with fluconazole, indifference was observed using the FICI method, whereas the Δ E method shows strong antagonistic effect (Table 3). This was evident in the plots as regions with negative ΔE values (Fig. 2). When ASA combined with caspofungin was used, the FICI method showed a synergistic action, while the Δ E method showed no relevant effect (Table 3), as ΔE values fluctuated across the tested concentrations without showing clear patterns of synergy or antagonism (Fig. 5). For combinations of ASA with itraconazole or terbinafine, the FICI values ranged between 0.5–4, and the ΔE values were close to zero, indicating no synergistic or antagonistic interactions (Figs. 3 and 7, Table 3). The contour and three-dimensional plots provide a detailed visualization of the drug concentrations that produce specific interactions, offering a comprehensive understanding of the effects (Figs. 2-7).

### Time–Growth Curve Analysis

**Fig. 8:**
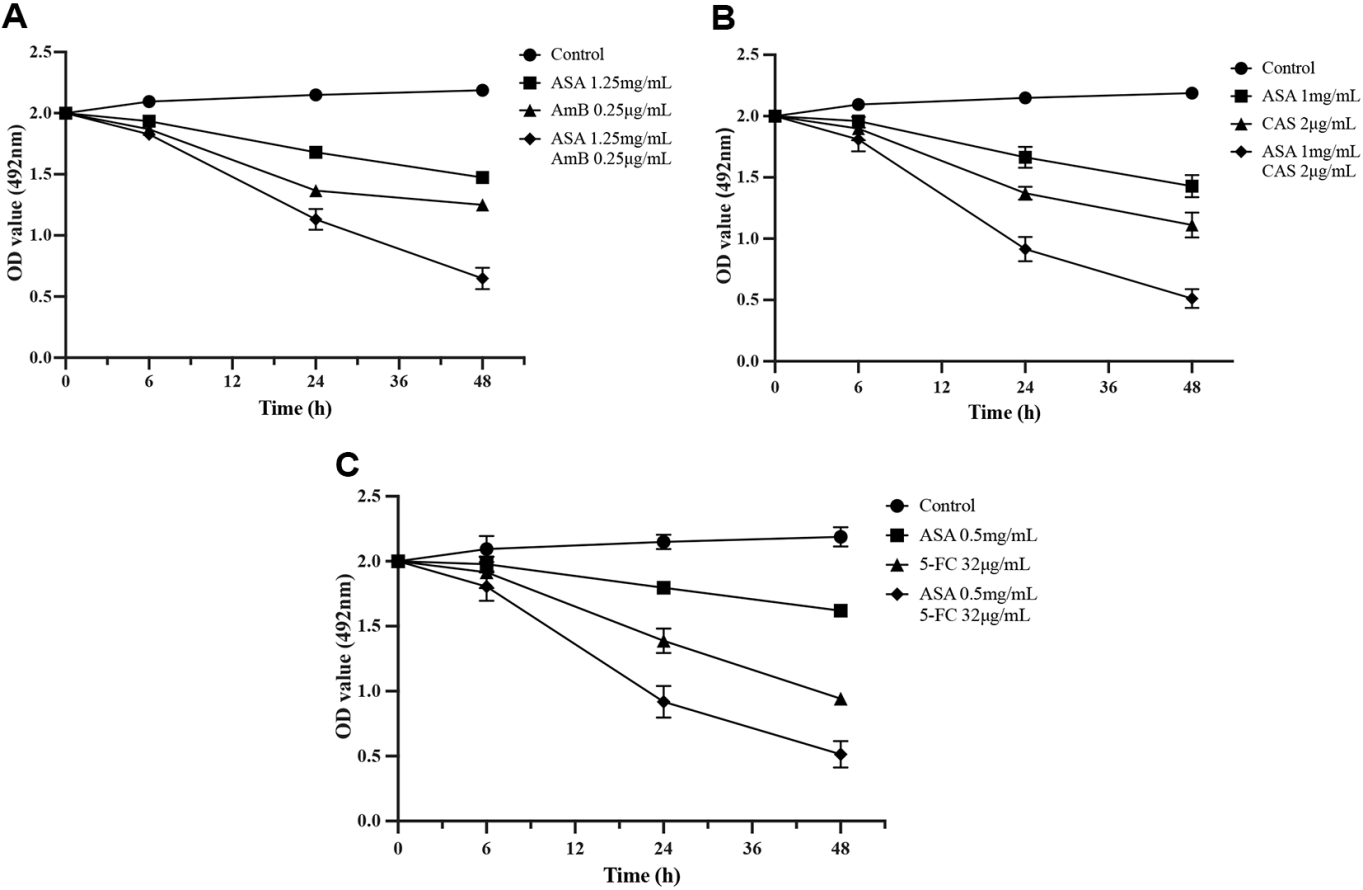
Time–growth curves of *C. albicans* biofilm. (**A**) treated with caspofungin (CAS) alone and combined with ASA; (**B**) treated with amphotericin B (AmB) alone and combined with ASA; (**C**) treated with 5-fluorocytosine (5-FC) alone and combined with ASA.

It was observed that under biofilm condition the SMIC_50_ of caspofungin was 64 μg/ml when treated with caspofungin alone, whilst the SMIC_50_ of caspofungin was decreased to 2 μg/ml after caspofungin combined with ASA. When amphotericin B was used alone, its SMIC_50_ was 2 μg/ml, whereas when combined with ASA was used, the SMIC_50_ of amphotericin B was reduced to 0.025 μg/ml. For 5-fluorocytosine, the SMIC_50_ was 128 μg/ml when used alone, and it decreased to 32 μg/ml when combined with ASA. As shown in Fig. 8, growth of *C. albicans* biofilm was inhibited by ASA alone, which was in line with the results of drug sensitivity. Compared with the ASA alone group, ASA combined with amphotericin B, 5-fluorocytosine or caspofungin further inhibit the growth of *C. albicans* biofilm (Fig. 8A-8C).

### ASA Increased the ROS Level of *C. albicans* Biofilm

**Fig. 9:**
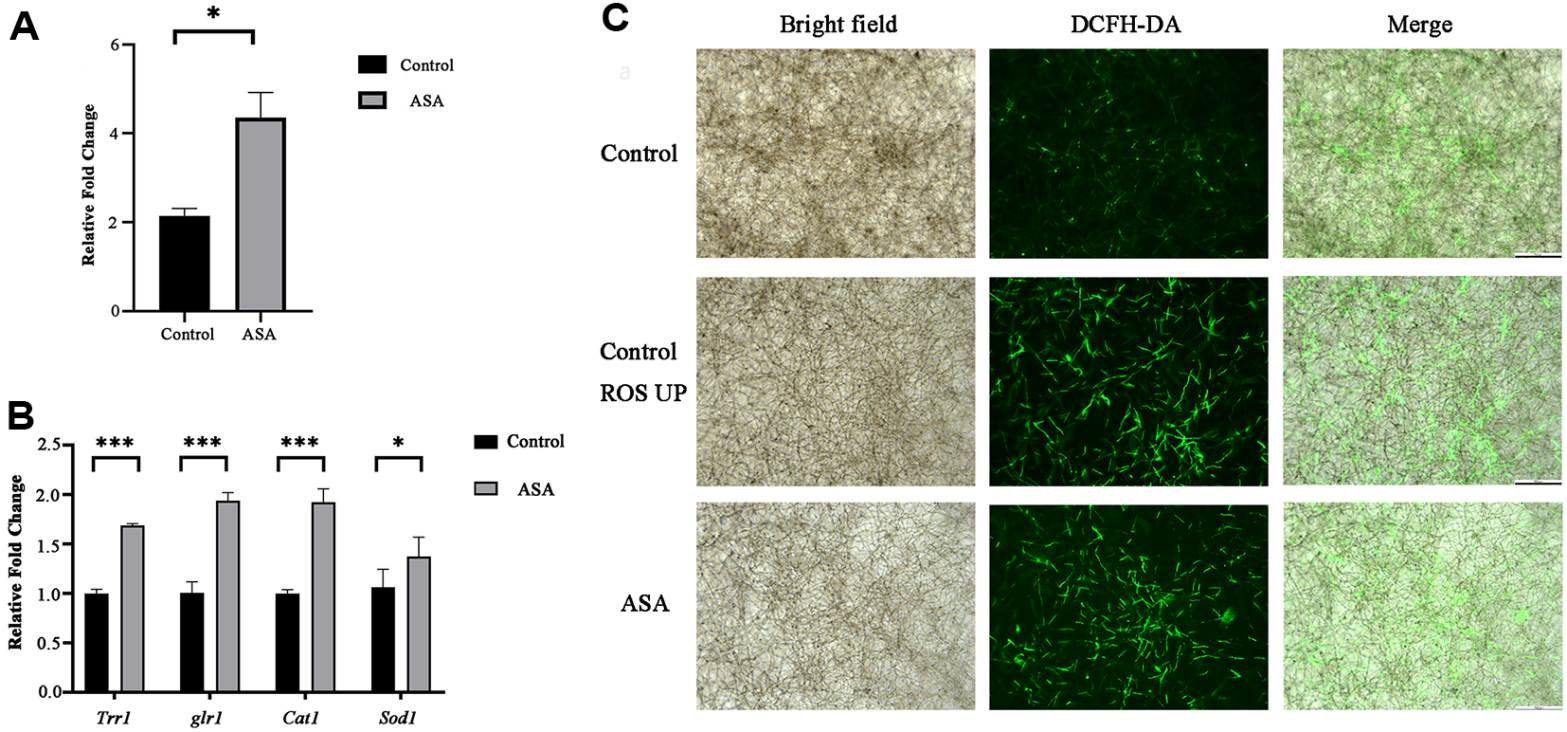
Oxidative damage in ASA-treated *C. albicans* biofilm. (**A**) Intracellular ROS were measured using DCFH-DA; (**B**) the expressions of ROS-related genes; (**C**) Representative DCFH-DA fluorescence images of *C. albicans* biofilm by inverted fluorescence microscopy. “ROS UP” refers to the increased levels of ROS under the experimental conditions used for comparison, representing a control condition where ROS levels are elevated.

Compared with control cells, ROS levels were significantly increased after incubation with ASA at the concentration of SMIC_50_ for 24 h (Fig. 9A). And the expressions of ROS-related genes (*Trr1, Glr1, Cat1*, and *Sod1*) were also up-regulated (Fig. 9B). Furthermore, significant fluorescence enhanced in the *C. albicans* biofilm after ASA treatment (Fig. 9C).

### ASA Induced Mitochondrial Dysfunction in *C. albicans* Biofilm

**Fig. 10:**
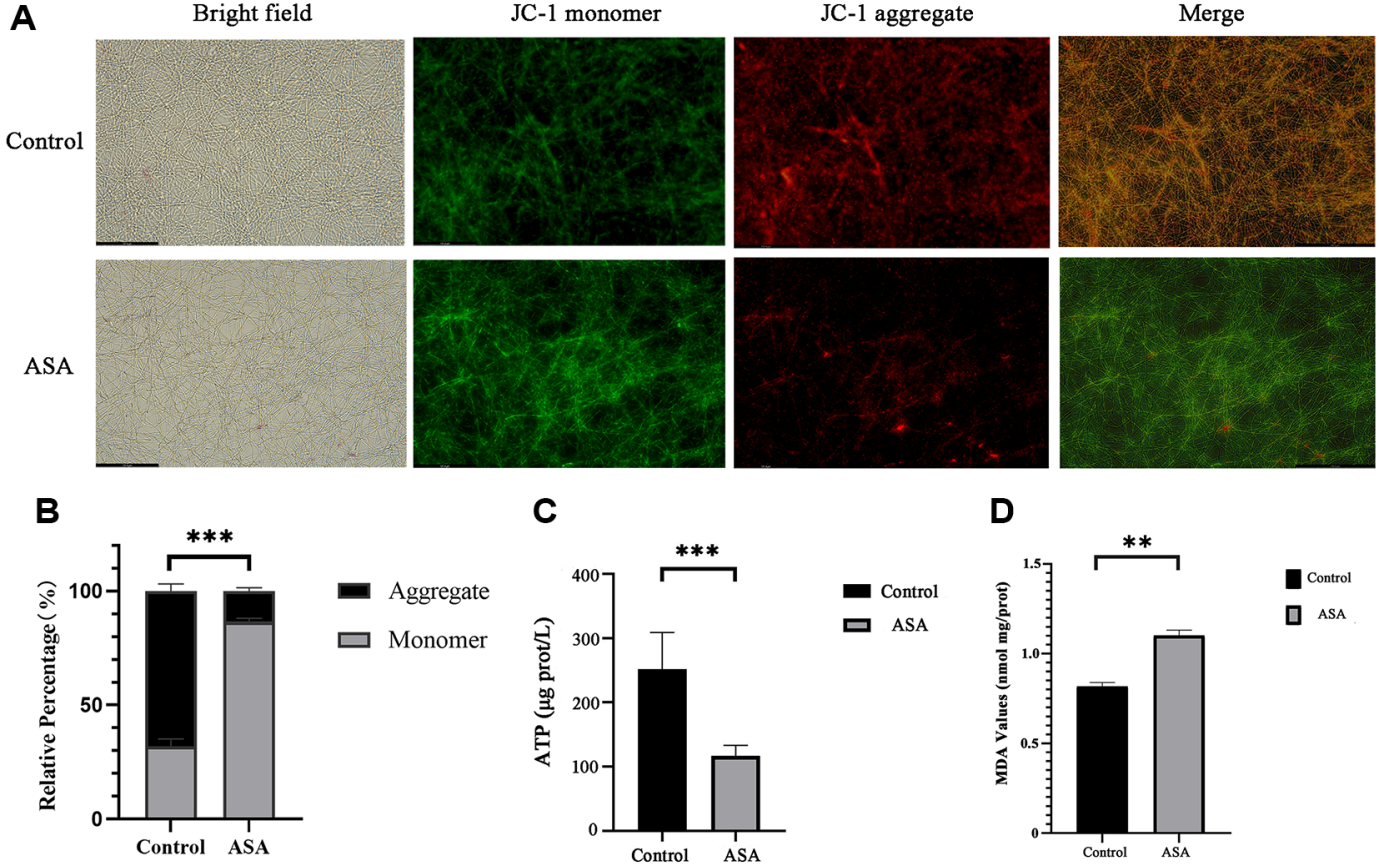
The effect of ASA on the mitochondrial membrane potential of *C. albicans* biofilm. (**A**) Typical JC-1 fluorescence images of *C. albicans* biofilm treated with ASA by inverted fluorescence microscope. JC-1 is a fluorescent dye that indicates mitochondrial membrane potential. Red fluorescence (aggregates) represents high membrane potential, while green fluorescence (monomers) represents low membrane potential; (**B**) The relative ratio of JC-1 red/green fluorescence of A analyzed by image J software; (**C**) The ATP level of *C. albicans* biofilm treated with ASA. (**D**) The MDA level of *C. albicans* biofilm treated with ASA.

Mitochondria is the main production workshop for ROS. However, excessive ROS can cause mitochondrial damage [19]. Therefore, this experiment further detected MMP and the intracellular ATP level to evaluate whether aspirin induced oxidative stress can lead to mitochondrial dysfunction in *C. albicans* biofilm. JC-1 is a potential-sensitive dye that exhibits a fluorescence shift depending on MMP. Healthy mitochondria with high MMP promote the aggregation of JC-1, resulting in red fluorescence, whereas depolarized mitochondria with low MMP keep JC-1 in its monomeric form, leading to green fluorescence [8]. The JC-1 fluorescence images showed that the green fluorescence intensity of ASA-treated *C. albicans* biofilm was greater than that of control (Fig. 10A). In addition, the relative ratio of JC-1 red/green fluorescence of ASA decreased significantly (Fig. 10B). The production of ATP and MDP is one of the important indicators for measuring mitochondrial function. A decrease in intracellular ATP levels and increase in MDA level indicates damage or decline in mitochondrial function [20, 21]. After treatment with ASA (4 mg/ml), the intracellular ATP of biofilms (24 h) significantly decreased, while the level of MDA increased when compared to the control (***p < 0.001) (Fig. 10C and 10D). These results indicated that ASA affects the integrity of *C. albicans* mitochondria, leading to mitochondrial membrane depolarization.

### ASA Inhibits mTOR Signaling and Induces Autophagy in *C. albicans* Biofilm

**Fig. 11:**
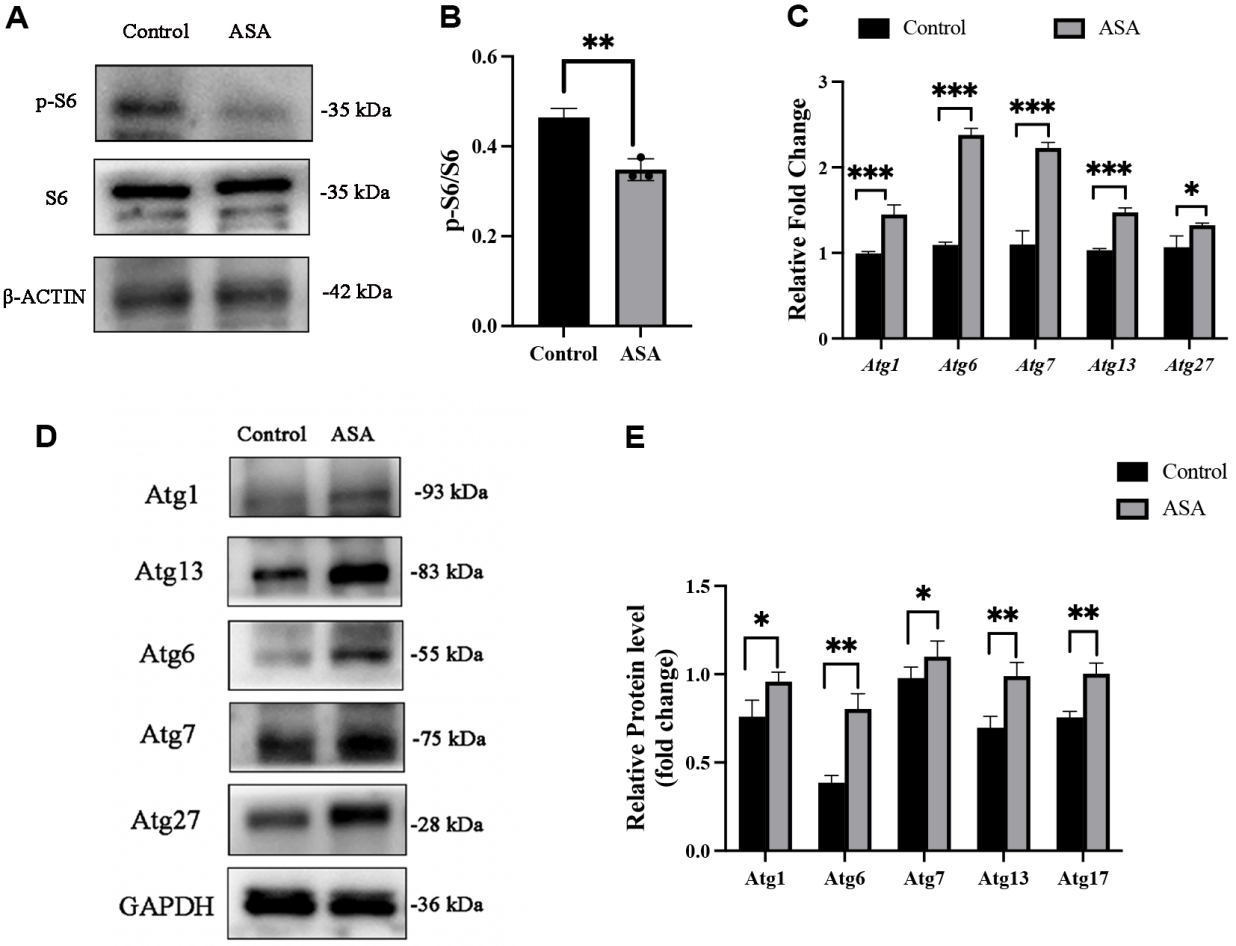
ASA inhibits mTOR and activates autophagy. (**A**) S6 ribosomal protein (S6) phosphorylation; (**B**) The quantitative protein analysis was performed for the western blot results in panel B; (**C**) The mRNA expressions of autophagyrelated genes in *C. albicans* biofilm treated with ASA; (**D**) The protein expressions of autophagy-related genes in *C. albicans* biofilm treated with ASA; (**E**) The quantitative protein analysis was performed for the western blot results in panel D. *: *p* < 0.05; **: *p* < 0.01; ***: *p* < 0.001.

**Fig. 12:**
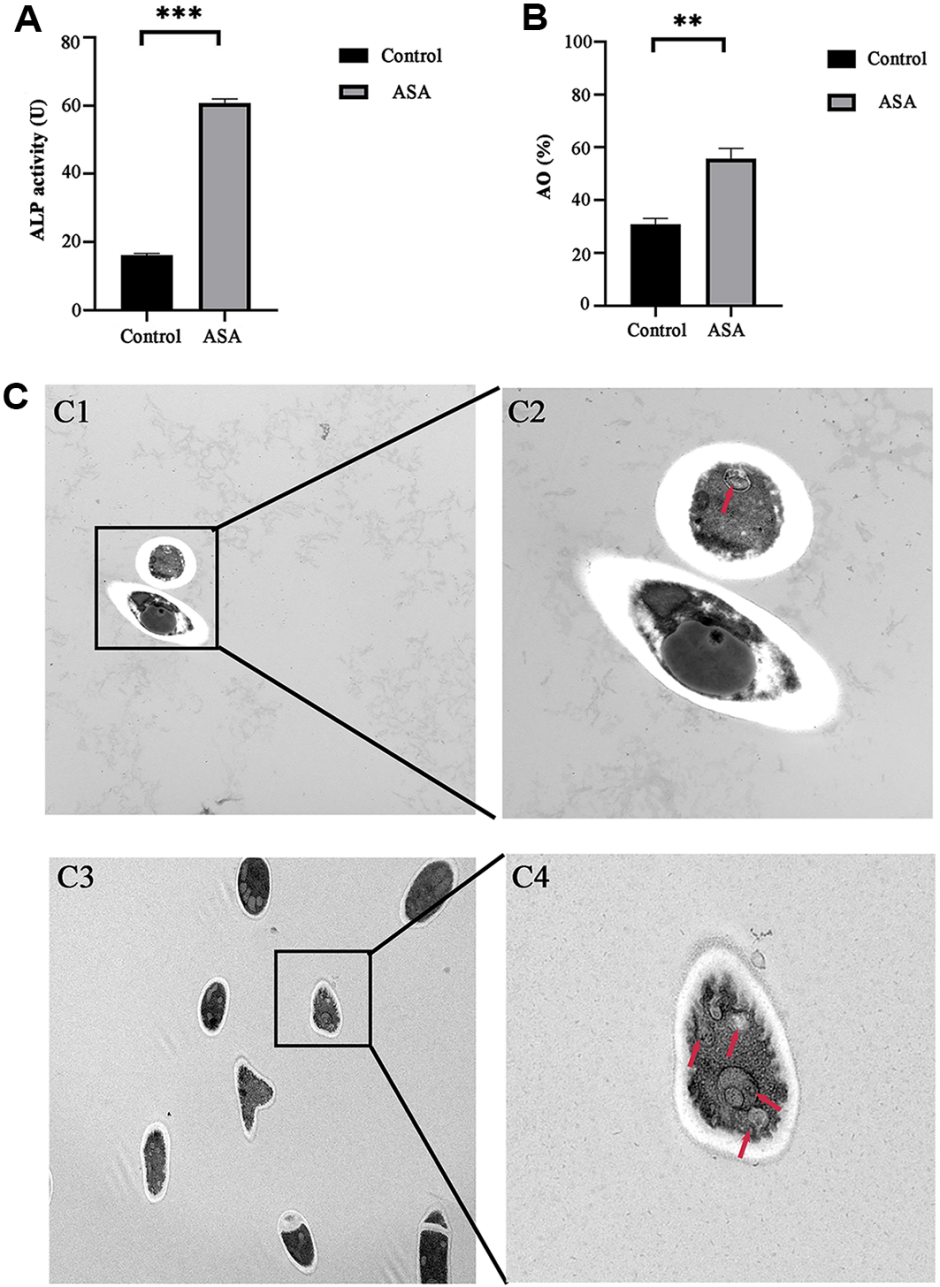
Autophagic activity of *C. albicans* biofilm after ASA treatment. (**A**) ALP activity in *C. albicans* biofilm; (**B**) The percentage of AO positive cells in *C. albicans* biofilm; (**C**) TEM imaging of *C. albicans* biofilm after ASA treatment, red arrow: autophagosome, (C1-C2) Control, (C3-C4) ASA, (C1 and C3) Scale bar: 2 μm, (C2 and C4) 500 nm. *: *p* < 0.05; **: *p* < 0.01; ***: *p* < 0.001.

The signal of phosphorylated ribosomal protein S6 (P-S6) is defined as a surrogate marker for mTOR dependent synthetic metabolic activity in *C. albicans* [22]. Therefore, the study investigated aspirin’s effects on the mTOR substrate S6 ribosomal protein (S6). After aspirin treatment, S6 phosphorylation was significantly reduced when compared with the control (Fig. 11A and 11B). Moreover, the mRNA expression of autophagy-related genes (*Atg1*, *Atg6*, *Atg7*, *Atg13*, *Atg17* and *Atg27*) were significantly increased in *C. albicans* biofilms treated with ASA when compared to the control (Fig. 11C) and the protein levels of autophagy-related genes in *C. albicans* biofilms were basically consistent to the mRNA expression levels (Fig. 11D and 11E). Autophagic activities were also detected by ALP activity and AO stain assay [8]. The ALP activity and the percentage of AO positive cells were both significantly up-regulated in ASA treated biofilm compared with the control group (Fig. 12A and 12B). Furthermore, TEM observation showed that less obvious autophagosome structure was observed in the control group, while more autophagosomes appeared in the biofilm treated with ASA, indicating autophagy occurred in cells when treated with ASA (Fig. 12C). These results suggest that aspirin has an inhibitory effect on mTORC1 signaling and induces autophagy in *C. albicans* biofilm.

## Discussion

Biofilm formation of *C. albicans* is intimately associated with its pathogenesis and development of resistance to conventional antifungal therapies [23]. The formation of biofilms as a cause of antimicrobial treatment failure requires the discovery and development of new antimicrobial therapeutic agents [24]. ASA, a well-known NSAID with analgesic effect, significantly decreases *C. albicans* biofilm formation and reduces the viability of biofilm cells at concentrations that could be achieved in humans with therapeutic doses, which limits its clinical application [3, 25]. However, ASA may be useful in combined therapy with conventional antifungal agents to reduce their dose and improve their efficacy. In this study, when treated with ASA combined with amphotericin B or 5-fluorocytosine, synergistic interactions were observed in *C. albicans* biofilms by the FICI and Δ E method and the SMIC_50_ of them were significantly reduced, indicating that ASA may act as an enhancer of the inhibitory effects of antifungal drugs on the growth of *C. albicans* biofilm. To assess the clinical feasibility of ASA-antifungal combination therapy, further studies on pharmacokinetics, toxicity, and dosing adjustments are necessary. ASA’s short half-life and gastrointestinal side effects, along with potential drug interactions, require careful consideration [12, 13]. For patients who suffer fungal infection meanwhile take ASA tablet as a commonly used medicine per day, combining it with antifungal agents like amphotericin B or 5-fluorocytosine might enhance treatment efficacy, reduce antifungal dosages, and minimize side effects and resistance. However, more research is needed to confirm its safety and effectiveness in clinical settings, particularly for chronic or drug-resistant infections.

Drug combination has been proved to be a valid and pragmatic strategy to seek drugs with novel mode of actions, which can potentially reduce the dose of single drug usage with increased drug-efficacy, and subsequently lower the drug toxicity [26]. Recent studies show that drug combinations, such as fluconazole with asiatic acid or ToAP2, improve Candida infection treatment by overcoming resistance and targeting multiple fungal pathways, offering enhanced efficacy with reduced toxicity [27, 28]. While the synergy between ASA and amphotericin B has been previously reported [29], the synergistic effect with 5-fluorocytosine has not. Amphotericin B has been shown to increase ROS levels in *C. albicans* cells [30], consistent with our findings that ASA also enhances ROS production in biofilms. Their combination might further enhance ROS, leading to oxidative stress and reduced biofilm cell survival. 5-fluorocytosine relies on cytosine osmotic enzymes to enter fungal cells and block protein synthesis and ASA has been proven to mediate membrane damage in *C. albicans* cells [31, 32]. PI staining experiments (Fig. S1) confirmed that ASA disrupts membrane integrity, as evidenced by increased fluorescence intensity in ASA-treated cells. The combination of ASA and 5-fluorocytosine caused the highest fluorescence, indicating enhanced membrane damage and increased permeability. These findings support the hypothesis that ASA facilitates the entry of 5-fluorocytosine into fungal cells, thereby boosting its antifungal efficacy. Furthermore, previous study showed that autophagy may play a role in the response to 5-fluorocytosine in *C. albicans* biofilms [8]. Although autophagy was not directly examined in relation to 5-fluorocytosine in our study, it may contribute to the enhanced efficacy observed. Further research is needed to clarify its role in the synergistic effect between ASA and 5-fluorocytosine in *C. albicans* biofilms.

Different results may be obtained by different methods [33]. In the study, when treated with ASA combined with caspofungin, FICI values suggested a synergistic effect, while the ΔE method indicated no significant interaction. This discrepancy can be attributed to the fact that the FICI method heavily relies on the MIC results, which may lead to inaccurate calculations when MIC exceeds the measurable range. In contrast, the Δ E model does not rely on the FICIs and MIC endpoints and is less sensitive to intraexperimental errors than the FICI model and it can complement the shortcomings of the FICI method. Although FICI is widely used due to its simplicity and feasibility, but it has limitations, particularly when MIC values are high or difficult to determine. ΔE, with its statistical approach and detailed visual representation, complements FICI and is particularly useful when MIC determination is challenging [29, 33].

The relationship between mitochondrial function, ROS production, and biofilm formation is complex. While low levels of ROS support cellular growth, excessive ROS from mitochondria, can inhibit biofilm formation by causing oxidative stress and cellular damage [30, 34]. Mitochondria are essential for energy production and biofilm resilience under stress [8, 19]. In this study, ASA significantly increased ROS levels and caused mitochondrial dysfunction in *C. albicans* biofilm cells, likely exceeding the threshold for self-regulation and leading to biofilm inhibition. The dysfunction may weaken the cells' ability to manage oxidative stress, reducing biofilm resilience and survival. Furthermore, ASA may also influence biofilm development by affecting hyphal growth, which is critical for biofilm formation and structural stability [1, 9]. SEM images revealed reduced hyphal structures and increased yeast-form cells in ASA-treated biofilms, suggesting that ASA disrupts biofilm architecture. These findings indicate that mitochondrial dysfunction, ROS overproduction, and impaired hyphal growth are key factors in biofilm inhibition. Further studies are needed to explore these mechanisms in detail.

Our results suggest that autophagy could regulate the biofilm formation of *C. albicans* and change the drug resistance of *C. albicans* biofilms to antifungal agents [8, 9]. The treatment with ASA can inhibit mTOR signaling and induce autophagic death in human tumor cells [12, 35]. Our study showed mTOR signaling was inhibited whereas autophagy was induced and activity was up-regulated after ASA treatment, indicating that this process is activated in *C. albicans* biofilm cells when treated with ASA. ASA has also been shown to induce mitochondrial dysfunction, reducing ATP production, and increasing ROS production in liver cancer, which was according and consistent with the results of ASA treatment of *C. albicans* biofilm in the study [36, 37]. Mitochondria are the main production workshop for ROS. However, excessive ROS can lead to mitochondrial damage [10, 19]. The interaction between ROS and autophagy is manifested by oxidative stress-induced autophagy and reduced accumulation of excessive ROS in autophagy [16]. In the study, the level of ROS increases, mitochondrial dysfunction occurs, and autophagy is activated after treat with ASA in *C. albicans* biofilms. Therefore, ASA might induce ROS accumulation and mitochondrial dysfunction and inhibit mTOR signal in biofilm cells, thereby activating autophagy and inhibiting *C. albicans* biofilm. While human tumor studies support ASA's mechanisms [36, 37], biofilms' unique features, such as their protective matrix and altered stress responses [1, 2], may influence ASA's effects. Further research is needed to determine if ASA's impact on biofilm formation applies to human biofilms in chronic fungal infections.

## Conclusion

Synergistic effects against biofilms were found for the combination of ASA and amphotericin B or 5-fluorocytosine. ASA activated autophagy by increasing oxidative stress and mitochondrial dysfunction and inhibited mTOR signaling, both actions suppress the *C. albicans* biofilm. These findings suggest that ASA combined with antifungal agents could be a promising strategy for biofilm-related fungal infections.

## Figures and Tables

**Table 1:** The sequences of primers for RT-*q*PCR.

Primer	Primer Sequences (5'→3')
*Atg1*-F	GTGGGTGATGGGCTTCT
*Atg1*-R	TGTGGTCGGTTGGTGC
*Atg6*-F	GCCAGAAATCAATGCCGCAT
*Atg6*-R	CCATCTACTGCATCCTTGGC
*Atg7*-F	CTGGGGTGTCAGGAGCATTA
*Atg7*-R	GCATCTACACCGGGGAAAAC
*Atg17*-F	CCATCGGAGTTCAAGCTTCC
*Atg17*-R	TCCGGTGATCATGTCCATCA
*Atg13*-F	AGTGTCCCGTCGTCTTCA
*Atg13*-R	ATGGAATCCTCATGACCCGT
*Atg27*-F	GCCACCTTCGCAAACAAA
*Atg27*-R	TGAAACCAAGCACCACCA
*Cat1*-F	CCAATTCCAGAACCATTTGCCACTC
*Cat1*-R	ACCATAAGCACCGGAACCTTTAGC
*Sod1*-F	TTGAACAAGAATCCGAATCC
*Sod1*-R	AGCCAATGACACCACAAGCAG
*Trr1*-F	TCTACGCCATTGGTCACATC
*Trr1*-R	ATCACCAGCTGCAAACACAC
*Glr1*-F	TGACAAGACTTTGATCGCCACTGG
*Glr1*-R	TCCAAGGCAAAGAACCCATCAGATG
*β-actin*-F	GACCAAGAAGACATCAAGGTATCAT
*β-actin*-R	GTGTTCAATTGGGTATCTCAAG

**Table 2:** Sensitivity of *C. albicans* biofilm to ASA and antifungal drug alone (μg/ml).

Drugs (μg/ml)	ASA	Fluconazole	Itraconazole	Amphotericin B	Caspofungin	5-Fluorocytosine	Terbinafine
SMIC_50_	4000	>128	>32	>2	>64	>128	>64

**Table 3:** In vitro interaction between ASA and antifungal drugs as determined by the FICI and Δ E method.

Drug combination (SMIC_50_)	FICI method		ΔE method (%)		
	Mean (range)	Interpretation	ΣSYN (n)	ΣANT (n)	Interpretation
ASA+fluconazole	1	IND	0 (0)	-1177 (49)	ANT (S)
ASA+itraconazole	1	IND	6 (8)	-98 (27)	IND
ASA+amphotericin B	0.4375	SYN	287 (49)	0 (0)	SYN (S)
ASA+caspofungin	0.5	SYN	54 (20)	-4 (9)	IND
ASA+5-fluorocytosine	0.375	SYN	325 (46)	-16 (5)	SYN (S)
ASA+terbinafine	1	IND	57 (14)	-68 (11)	IND

SYN: synergism; ANT: antagonism; IND: indifference. (S) strong synergism or antagonism.
